# School nursing in children's health from a holistic perspective

**DOI:** 10.15649/cuidarte.4668

**Published:** 2025-09-01

**Authors:** Ivone Tatiana Brito Jiménez, Julieth Karina Brito Jiménez, Carolina Elena Cortina Navarro

**Affiliations:** 1 Universidad del Magdalena. Facultad Ciencias de la Salud. Santa Marta, Colombia. E-mail: ibrito@unimagdalena.edu.co Universidad del Magdalena Santa Marta Colombia ibrito@unimagdalena.edu.co; 2 Universidad del Magdalena. Facultad Ciencias de la Salud. Santa Marta, Colombia. E-mail: ybrito@unimagdalena.edu.co Universidad del Magdalena Santa Marta Colombia ybrito@unimagdalena.edu.co; 3 Universidad del Magdalena. Facultad Ciencias de la Salud. Santa Marta, Colombia. E-mail: ccortina@unimagdalena.edu.co Universidad del Magdalena Santa Marta Colombia ccortina@unimagdalena.edu.co

**Figure 1:**
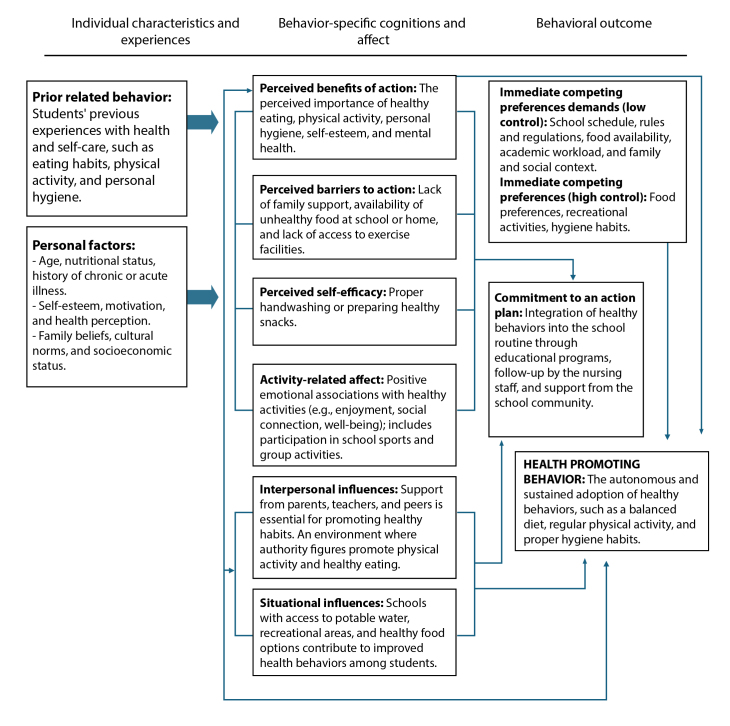
Adaptation of Nola Pender’s Health Promotion Model to the school nursing context

*Source: Adapted from Nola Pender's Health Promotion Model, 1996^2^*

School nursing plays an essential role in promoting health and improving the quality of life for children and adolescents. This role is not limited to direct care but extends beyond into health education, disease prevention, and student empowerment through early interventions and health promotion strategies. Within this context, Nola Pender's Health Promotion Model proposal^1^ serves as an essential guide for school nurses' actions. This model considers individual, social, and environmental factors that influence health behaviors, encouraging informed and healthy choices from early childhood^2^, as shown in the Figure 1 below.

In educational settings, services should be provided that promote individual and collective skills within the school community, with the aim of preventing, identifying, and addressing health issues^3^.

Training of nursing students within school environments, through instruction and guidance, contributes significantly to their well-being, promotes their retention in professional practice, and enhances their performance^4^. By equipping them with specific knowledge about healthy habits, not only is their performance improved, but the necessary competencies to face the challenges of the educational setting are strengthened. This, in turn, positively impacts student well-being and enhances the overall quality of educational institutions and the school environment.

School nursing experts identify the need for empirical studies that establish a link between school nursing actions and student academic performance^5^. It is important to have empirical evidence of how nursing interventions within educational contexts directly influence students' academic performance. Studies that connect the physical and emotional well-being of students, fostered through nursing interventions, to academic achievement are essential, as this relationship remains insufficiently documented.

In recent years, nursing students from the Universidad del Magdalena have promoted healthy lifestyles through health education, with a focus on strengthening this field of practice. In addition, they work on the development of life skills, such as stress management and conflict resolution, contributing to the creation of a safe, inclusive, and supportive school environment for learning, with the aim of prioritizing students' comprehensive well-being.

Martinez et al.^6^ describe the roles of nurses in delivering school health services, noting that school nurses must not only address students' health needs but also provide assistance to students' families, staff, and the community. Health professionals have a responsibility to promote and reinforce healthy lifestyles, paying special attention to healthcare from the early stages of life. This effort should begin within the family and extend into the school setting, with the aim of promoting a culture of health that benefits both individuals and the community, as health is a fundamental resource for collective well-being^7^.

**Regulatory framework and global strategies in school nursing**

The Sustainable Development Goals (SDGs), especially SDG 3: “Ensure healthy lives and promote well-being for all at all ages,”^8^ highlight the critical role of school nurses in achieving these objectives by delivering health education, offering emotional support, and coordinating services for both healthy students and those with chronic or complex health needs. In addition, school nurses contribute to reducing health disparities, aligned with SDG 10, by ensuring that all students, regardless of socioeconomic background, have access to quality health services^9^.

Social determinants of health, including poverty, education, access to healthcare services, and the social and physical environment, have a significant impact on children's development and well-being. School nurses address these determinants by acting as liaisons between the school, families, and community resources, and by intervening early in health issues that could hinder students’ academic achievement or impact their social and emotional development^10^.

The Comprehensive Care Route (Ruta Integral de Atención, RIA) addresses actions targeted at early childhood and infancy. Within this framework, school nursing is essential to provide continuous and holistic care to children. This involves not only managing health conditions but also promoting healthy lifestyles and developing skills from an early age^11^. Based on Nola Pender's model, school nursing supports a culture of preventive care by fostering self-efficacy and informed decision making^12^.

The impact of school nursing within the educational community is significant, benefiting not only students but also teachers and school staff. School nurses play a key role in health promotion, disease prevention, and early intervention, which, in turn, improves the overall educational environment.

The presence of school nurses reduces absenteeism, facilitates actions such as early detection of mental health issues, stress management, and bullying prevention, and improves care for students with chronic conditions, which has a direct impact on their academic performance and integration into the school setting^13^. The mental health of children and adolescents is a growing priority, requiring concrete tactics within the context of educational nursing^14^.

According to the report by a World Health Organization (WHO) working group, nurses represent the first and strongest link between the health system and individuals^15^. They can identify needs, deliver care, and play a crucial role in health education for children, whether healthy or clinically ill, as well as for their parents and the broader educational community^16^.

The school nurse ensures safety within the educational environment by preventing the teaching staff from having to assume responsibilities related to specific care or interventions in emergency situations^17^. Moreover, the emotional support provided by school nurses is essential to student well-being, as it enables the early detection of mental health concerns and facilitates referrals to specialized healthcare services^13^. In addition, school nurses are on the front lines of school safety, managing medical emergencies, overseeing the administration of medications, and preventing outbreaks of infectious diseases^18^.

The relevance of the role of school nursing in promoting child health underscores the need to create public policies that strengthen and expand its presence in schools. These policies should focus on strengthening the academic training of nursing professionals in this area, guaranteeing an adequate number of nurses per institution, providing resources for continuing education, and defining nurses' roles in the comprehensive care of students. Given the positive impact of school nursing on the physical, emotional, and social well-being of children, as well as its contribution to creating safer and healthier school environments, it is essential that governments prioritize the inclusion of school nurses as a key part of public health and educational strategies. This approach ensures that both preventive and emergency health interventions are integrated into long-term educational programs.

In conclusion, the role of the school nurse is multifaceted and essential for promoting child health. By integrating theoretical models, such as Nola Pender's, aligning with the Sustainable Development Goals (SDGs), and considering the social determinants of health, school nursing offers a comprehensive and effective approach to improve the health and well-being of children and adolescents in educational settings.

Within the framework of the Universidad del Magdalena's mission, the students of the Nursing Program emphasize the importance of their role through their commitment to promoting healthy lifestyles via health education activities in school environments. Moreover, they are encouraged to actively participate in the improvement of public policies that strengthen comprehensive school health care, thereby contributing to the creation of healthier, more inclusive, and sustainable educational communities.
